# Palatal positioned implants in severely atrophic maxillae versus conventional implants to support fixed full-arch prostheses: Controlled retrospective study with 5 years of follow-up

**DOI:** 10.4317/medoral.20262

**Published:** 2015-02-07

**Authors:** Eugenia Candel-Marti, David Peñarrocha-Oltra, Leticia Bagán, Maria Peñarrocha-Diago, Miguel Peñarrocha-Diago

**Affiliations:** 1 Collaborating Professor of the Master in Oral Surgery and Implantology, Stomatology Department, Faculty of Medicine and Dentistry, University of Valencia, Valencia, Spain; 2 Collaborating Lecturer in Oral Medicine, Dental Medicine Division, Stomatology Department, Faculty of Medicine and Dentistry, University of Valencia, Valencia, Spain; 3 Full Professor of Oral Surgery, Stomatology Department, Faculty of Medicine and Dentistry, University of Valencia, Valencia, Spain; 4 Chairman of Oral Surgery, Director of the Master in Oral Surgery and Implantology, Stomatology Department, Faculty of Medicine and Dentistry, University of Valencia, Valencia, Spain

## Abstract

**Background:**

To evaluate soft tissue conditions and bone loss around palatal positioned implants supporting fixed full-arch prostheses to rehabilitate edentulous maxillae with horizontal atrophy and compare them with conventional well-centered implants placed in non-atrophic maxillae after a minimum follow-up of 5 years.

**Material and Methods:**

A clinical retrospective study was performed of patients that were rehabilitated with full-arch fixed implant-supported maxillary prostheses and had a minimum follow-up of 5 years after implant loading. Patients were divided into 2 groups: patients with class IV maxilla according to Cawood and Howell and treated with palatal positioned implants (test) and with class III maxilla and treated with implants well-centered in the alveolar ridge and completely surrounded by bone (control). The following variables were assessed: age, sex, frequency of tooth brushing, smoking, type of prosthesis, type of implant, implant success, amount of buccal keratinized mucosa, buccal retraction, probing depth, plaque index, modified bleeding index, presence of mucositis or peri-implantitis and peri-implant bone loss. Statistical analysis was performed applying Chi2 Test and Student’s t-test using alpha set at 0.05.

**Results:**

A total of 57 patients were included: 32 patients with 161 palatal positioned implants (test) and 25 patients with 132 well centered implants (control). No statistically significant differences were found regarding age, sex and smoking, but test group patients reported a significantly higher frequency of daily tooth brushing. Implant success rates were 96.9% for test group implants and 96.0% for control group implants. Peri-implant mucosa retraction was significantly higher in the control group than in the test group (*p*=0,017). No significant differences were observed either for all the other assessed clinical parameters or for peri-implant bone loss.

**Conclusions:**

Despite its limitations the outcomes of the present study suggest that palatal positioned implants may be a good treatment alternative for patients with severe horizontal maxillary alveolar bone atrophy. Palatal positioned implants presented similar success rates, soft tissue conditions and peri-implant bone loss than well-centered implants placed completely surrounded by bone in non-atrophic ridges.

**Key words:** Atrophic maxilla, bone atrophy, fixed dental prosthesis, dental implants.

## Introduction

According to the original protocol by Brånemark, dental implants should be placed upright, centered in the bone crest and completely surrounded by bone (1). This position can only be achieved in class III maxillae according to Cawood and Howell (2); i.e., maxillae with enough bone height and width. In class IV maxillae, where there is sufficient bone height but insufficient bone width, placement of dental implants completely surrounded by bone is complicated (3). To resolve or bypass this situation, numerous surgical techniques have been proposed. These methods can be classified into bone grafting techniques (i.e., guided bone regeneration or block grafts (4) and modifications of the original implant insertion protocol that avoid bone grafting by using areas of residual bone (i.e., zygomatic implants (5), pterygoid implants (6), implant insertion in the maxillary tuberosity (7) and tilted implants) (8).

The use of bone grafting to allow implant placement in atrophic maxillae is associated with more frequent complications, higher morbidity, increased economic costs and a longer treatment time than the placement of conventional implants in non-atrohpic maxillae (3). An alternative to bone grafting in maxillae with a narrow residual crest (width < 4 mm) is the insertion of implants in a palatal position (7,8-10). This modification allows to have 2 mm of buccal bone even in atrophic ridges, while 2 to 5 implant threads are left exposed and covered with particulate bone graft (11).

The aim of the present study was to evaluate soft tissue conditions and bone loss around palatal positioned implants supporting fixed full-arch prostheses to rehabilitate edentulous maxillae with horizontal atrophy and compare them with conventional well-centered implants placed in non-atrophic maxillae after a mínimum follow-up of 5 years.

## Material and Method

* Study design

A clinical controlled retrospective study was performed in the Oral Surgery and Implant Dentistry Division of the University of Valencia January and December 2013. The research was performed following the principles of the Declaration of Helsinki on research involving human beings. Accordingly, all patients were informed about the study and they were asked to sign an informed consent document before being included. The study design was approved by the ethical review board of the University of Valencia (Ref: H1330446292077).

A chart-review was performed to retrospectively select patients according to the following criteria.

Inclusion criteria:

- Rehabilitation of the edentulous maxillae with fixed implant-supported prosthesis

- No previous bone grafting procedure to reconstruct atrophic alveolar ridges and allow implant insertion

- Minimum follow-up of 5 years after implant loading

Exclusion criteria:

- Failure to attend scheduled control visits

- Refereed patients not being controlled at the Oral Surgery and Implant Dentistry Division

- Patients not agreeing to participate in the study

Included patients were divided into 2 study groups.

- Test group: patients with class IV maxilla according to Cawood and Howell (2) and treated with palatal positioned implants in the anterior and premolar regions. Implants placed in molar regions were excluded as bone atrophy at this level is mainly vertical so implants are well-centered in the alveolar crest even in atrophic cases.

- Control group: patients with class III maxilla according to Cawood and Howell (2) and treated with implants well-centered in the alveolar ridge and completely surrounded by bone. In order to maintain both groups as homogeneous as posible implants placed in molar regions were also excluded from the control group.

* Surgical protocol

A clinical and radiographic examination was performed of all the patients, including panoramic radiography and computed tomography for surgical planning. The minimum amount of bone for implant placement was 8 mm in height and 3 mm in width (measured at crestal level). All surgeries were performed by the same surgeon under local anesthesia with articaine 4% with epinephrine 1:100,000 (Inibsa, Lliça of Vall, Barcelona, Spain) and / or sedation with propofol solution of 1%; blood pressure, pulse and oxygen monitoring was performed by an anesthesiologist. All implants were Phibo® TSA with Avantblast Surface (Phibo Dental Solutions, Senmenat, Barcelona, Spain).

**Figure 1:**
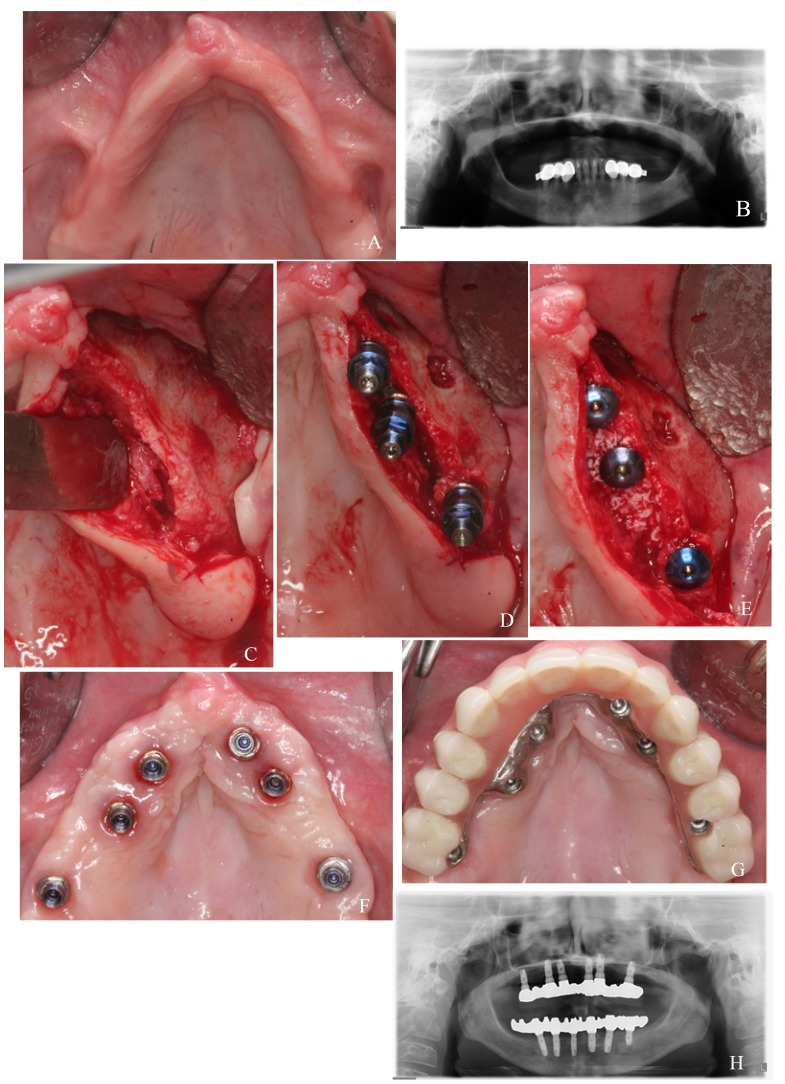
Test group case: a) preoperative clinical image; b) preoperative panoramic radiograph; c) narrow alveolar ridge of the second quadrant; d) palatal positioned implants in canine and premolar positions; e) particulate bone graft covering exposed threads in the palatal side; f) healed soft tissues; g) placement of the metal-resin screwed prosthesis; h) panoramic radiograph after 5 years of follow-up.

Test group: Surgical procedures for the rehabilitation of atrophic maxillae with palatal positioned implants were detailed in a previous report (11). Implant sites were prepared combining drills and osteotomes to conserve as much bone as posible. Implants in the anterior and premolar regions were placed in palatal position, with 2 to 5 threads exposed on the palatal side. Exposed thread were covered with autologous particulate bone (when available) and Bio-Oss (Geistlich, Wolhusen, Switzerland). In molar regions the bone crest generally has sufficient width to place implants completely surrounded by bone. Implants were left submerged during 3 months (Fig. 1a-h ).

**Figure 2:**
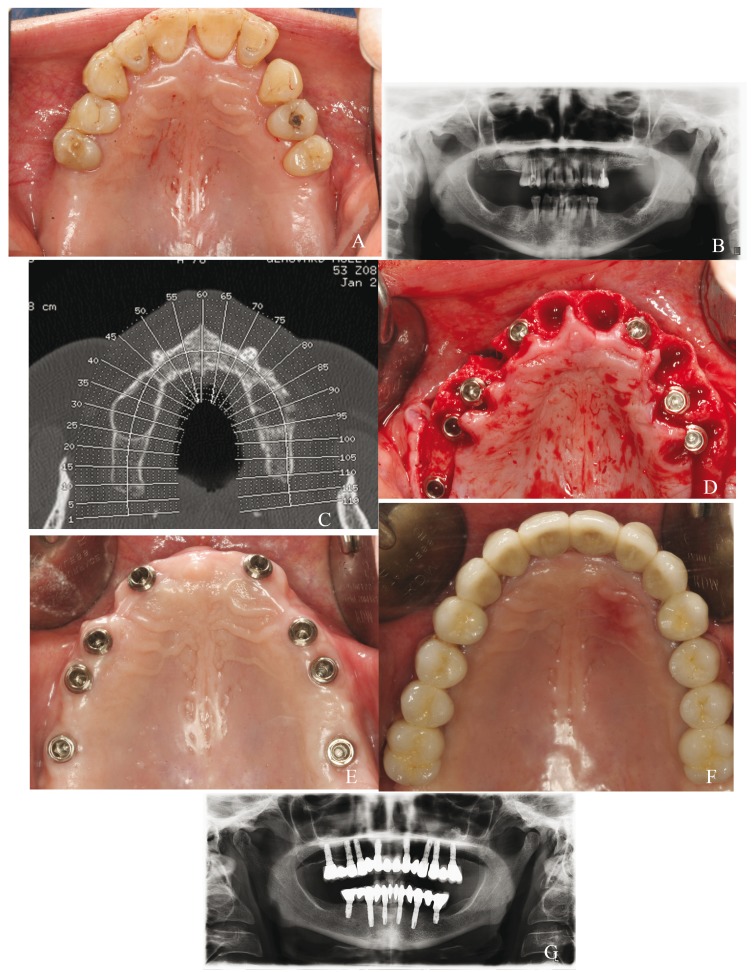
Control group case: a) preoperative clinical image; b) preoperative panoramic radiograph; c) CBCT scan to study bone availability; d) placement of 8 post-extraction implants, well-centered in the alveolar crest; e) healed soft tissues 1 months after the surgery; f) metal-ceramic fixed prosthesis; g) panoramic radiograph taken after 5 years of follow-up.

Control group: Implants were placed well-centered in the alveolar ridge and completely surrounded by bone. Sites were prepared combining drills and osteotomes and implants were left submerged during 3 months (Fig. 2 a-g).

* Prosthetic procedures

Fixed metal-ceramic prostheses were placed when the interoclusal space, the inter maxillary relation and the patient’s lip support were adequate. Screwed metal-resin prostheses were used when the interocclusal space was excessive or to compensate for lack of lip support.

* Follow-up and maintenance

Sutures were removed 1 week after the surgery. Prosthesis fabrication began 3 months after implant placement. All patients were included in a maintenance program with controls visits involving profesional prophylaxis every 6 months.

* Data gathering

All data collection was made by a single trained clinician, different from the surgeon or the prosthodontist, following a pre-established protocol.

**Figure 3:**
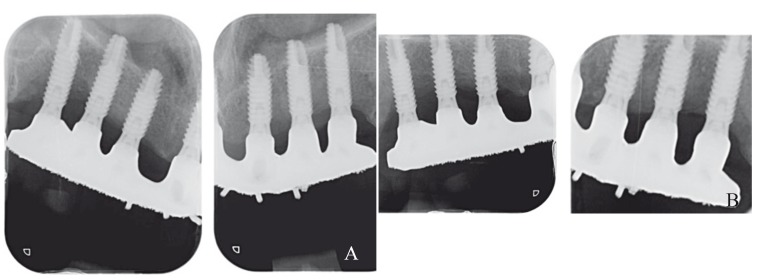
a, b) Periapical radiograph taken at (a) implant loading and (b) the last control visit (6 years post-loading).

- General variables: age, sex, frequency of tooth brushing (0-3 times/day), smoking (number of cigarettes / day), type of prosthesis (metal-ceramic / metal-resin) and type of implant (immediate post-extraction / placed in healed bone) were registered.

- Implant success: success was evaluated according the criteria defined by Buser *et al*. (12): 1) absence of clinically detectable implant mobility; 2) absence of pain or any subjective sensation; 3) absence of recurrent peri-implant infection and 4) absence of ongoing radio lucency around the implant after six and twelve months of loading.

- Periimplant soft tissue stability: The amount of buccal keratinized mucosa, buccal retraction and probing depth (PPD; at 6 points) were evaluated using a millimitred probe. Plaque index (PI- range 0-3) and modified bleeding index (BI- rango 0-3) were assessed according to Mombelli *et al*., (13) criteria. Based on the Consensus Report of the VI European Workshop on Period ontology (14), implants with peri-implant mucosal redness, swelling, bleeding on probing, and without radiographic signs of bone loss were considered to present peri-implant mucositis. Those implants in which the soft tissue lesion was associated with marginal bone loss and sometimes with suppuration and/or increased probing depth were considered to present peri-implantitis.

- Peri-implant bone loss: Periapical radiographs obtained at prosthesis placement and after at least 5 years were used to calculate bone loss. Radiographs were obtained with the XMIND® intraoral system (Groupe Satelec-Pierre Rolland, Merignac, France) and an RVG intraoral digital receptor (Dürr Dental, Bietigheim-Bissingen, Germany). Periapical radiographs were made using the paralleling technique with a film holder and an aiming device (Rinn XCP®, DentsplyRinn, Elgin, IL, U.S.A). If the bone level around the study implants was not clearly visible a new radiograph was made. Peri-implant marginal bone levels were measured by the same operator using Cliniview® 5.1 software (Instrumentarium Imaging, Tuusula, Finland). Each image was calibrated using the known diameter of the implants. The vertical distance from the outer edge of the implant shoulder (reference point) to the most coronal bone-to-implant contact was measured to the nearest 0.1 mm. Peri-implant marginal bone resorption at the mesial and distal aspect of the implants was calculated from the change in bone level between the baseline and the 1-year control radiograph; for each pair of measurements the largest value was used (15) (Fig. 3).

* Statistical analysis

A descriptive analysis was performed of the registered variables. To study differences between the groups, a comparative analysis was performed applying Chi2 Test for categorical variables and Student’s t-test for continuous variables. Statistical analysis was performed using SPSS 15.0 software (SPSS Inc., Chicago, IL) using an alpha value of 0.05. A biostatistician with expertise in dentistry analyzed the data without knowing the group assignment.

## Results

The chart review yielded 66 patients with 457 implants fulfilling the inclusion criteria. Nine patients were excluded: 4 failed to attend scheduled control visits and 5 were refereed patients. A total of 57 patients - 32 belonging to test group and 25 to the control group - were finally included. The mean follow-up was 6.5 ± 1.3 years (range 5-11).

Patients from the test group received 225 implants. 161 were palatal positioned and were included and 64 were excluded: 47 were placed well-centered in molar regions, 9 were pterigoid implants and 8 were zygomatic implants. The mean age in this group was 55 ± 10.5 years and 75% of the patients were women. Patients from the control groups received 182 implants, all of them well-centered in the alveolar crest. The mean age in this group was 55,9 ± 7,9 years and 48% of the patients were women. Fifty were placed in molar regions and thus excluded. Out of the 132 included control group implants 30 were immediate post-extraction.

The patient sample was homogeneous regarding age, sex and smoking habit. Significant differences were observed in frequency of tooth brushing, being the mean value higher in the test group. Descriptive statistics for smoking habit and frequency of tooth brushing is detailed in  Table 1.

**Table 1:**
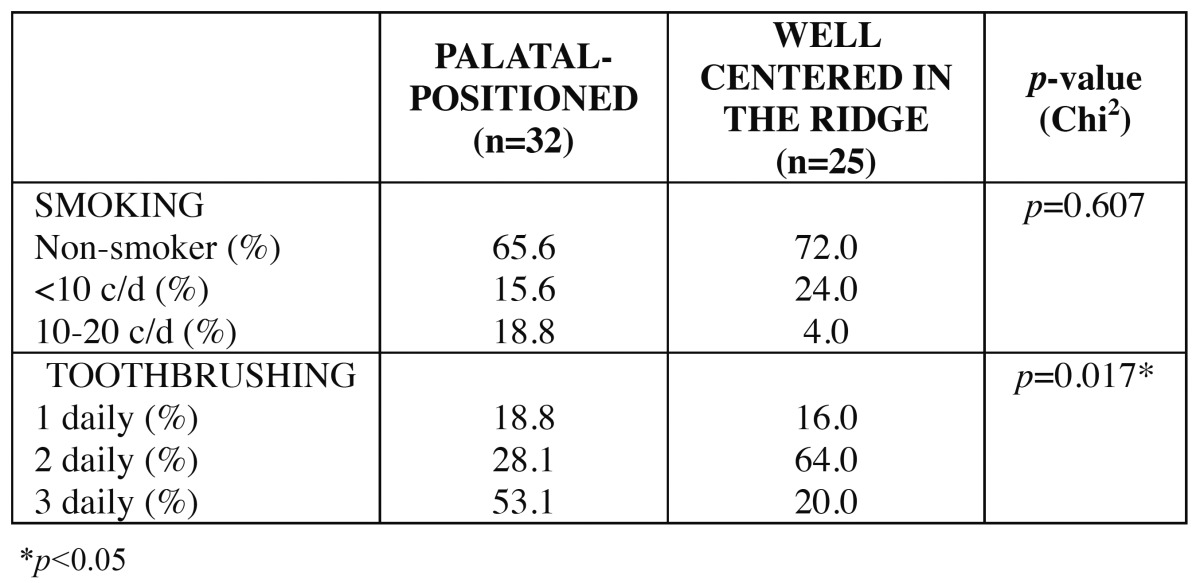
Statistics for smoking and frequency of tooth brushing.

In the test group all the patients received metal-resin screwed prostheses, while in the control group 40% of the prostheses were metal-ceramic and 60% were metal-resin.

One implant was lost in the test group and another one in the control group. The success rates were 96.9% and 96.0% respectively and differences were non-significant.

Results regarding peri-implant soft tissues conditions are described in  Table 2. Significant differences were only observed for periimplant mucosa retraction (*p*=0.017), being values higher for the control group.

**Table 2:**
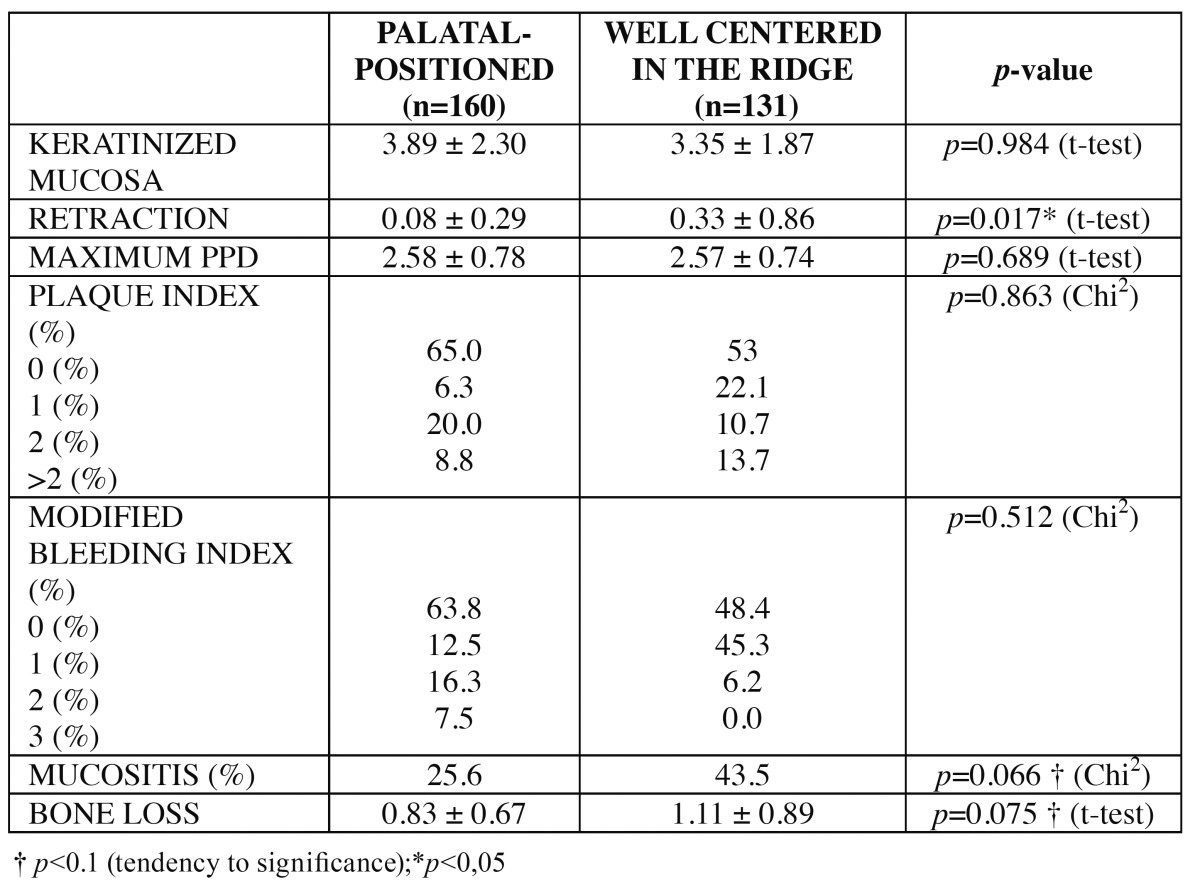
Statistics for clinical peri-implant parameters.

A peri-implant bone loss of 0.83 ± 0.67 mm was calculated for the test group and of 1.11 ± 0.89 mm for the control group; differences were non-significant ( Table 2).

## Discussion

In patients with horizontal alveolar maxillary bone atrophy implants cannot be placed in a standard manner. The most common alternative to allow rehabilitation using implants is to perform bone grafts to increase the width of the bone ridge prior to implant placement. In severe atrophies the amount of bone that can be harvested from intraoral locations is not sufficient and bone grafts from extra oral areas require hospitalization, have higher economic costs and increased donor site morbidity (including pain, neurosensory deficits and functional limitations). Furthermore, the grafted bone resorption is common and unpredictable (16).

Placing implants in a palatal position is an alternative in cases of horizontal alveolar bone atrophy (7). This technique enables rehabilitating atrophic patients with similar costs and morbidity to those of conventional implants (well-centered in the alveolar ridge) used in patients with sufficient bone, although it has been scarcely studied in the Literature. For this reason a controlled study was planned, to analyze the clinical and radiologic outcome of these implants after a minimum medium-term follow-up (5 years) and to compare them with conventional implants. The next step after this retrospective and controlled but nonrandomized study should be prospective randomized controlled trials to compare this technique with other alternatives for the rehabilitation of atrophic maxillae. These future studies should consider evaluating implant and prosthetic success, morbidity, treatment time and patient satisfaction and quality of life.

Success rates for palatal positioned and conventional implants resulted to be similar (96.9% and 96.0% respectively) in the present study. In 1999, Mattson *et al*. (7) performed a study in which dental implants were placed in maxillae with class V or VI according to Cawood and Howell (2) without involvement of bone grafting techniques. Similarly to the present study, implants were anchored in the palatal bone plate and left with 2 to 5 exposed threads in the palatal side. These authors reported favorable outcomes for both soft and hard tissues with these implants, and a 99% success rate after a mean follow-up of 3.75 years. Rosen and Gynther (15) rehabilitated 19 patients with atrophic maxillae using 103 implants that were left with exposed threads in the palatal side; a 97% success rate was reported after 8 to 12 months of follow-up.

Few studies have assessed soft-tissue clinical parameters around palatal positioned implants. In the present study, palatal positioned implants presented healthier soft tissues than conventional implants, although differences were only significant for mucosal retraction. This may explained by the conservation of an intact wide buccal bone plate when implants are placed in a palatal position (17). The presence of 30 immediate post-extraction implants in the control group could also be related with this difference in retraction. Moreover, test group patients had higher mean frequency of daily tooth brushing. These patients are generally particularly motivated with maintenance and oral hygiene, as they are conscious of the complexity of their case due to the severe lack of bone. 25.6% of the test group implants and 43.5% of the control group implants presented with peri-implant mucositis; no implant had peri-implantitis. Rosen and Gynther (15) observed mucositis in 9 out of 19 patients treated with palatal positioned implants and no peri-implantitis. These authors suggested that mucositis could be associated with por oral hygiene. Leckholm *et al*. (18), reported that implants placed with some exposed threads in the palatal side did not show more soft tissue complications than implants completely surrounded by bone.

Peri-implant bone loss was 0.83 ± 0.67 in the test group and 1.11 ± 0.89 mm in the control group. Rosen and Gynther (15) found a mean bone loss of 1.2 mm in 5 patients after 8 to 12 years of follow-up and no bone loss in the other 14 patients treated with palatal positioned implants. Mattson *et al*. (7) followed 86 implants for 1 to 3 years and none of them presented > 1 mm of bone loss. The presence of a well preserved and thick buccal bone plate has been related with reduced bone loss around post-extraction implants. The same principle may explain the favorable results of palatal positioned implants regarding bone loss. In the present study and in all the reviewed articles on patalal positioned implants bone was evaluated using paralleled periapical radiographs. This provides only 2D information, and the thick buccal bone is probably hiding in some cases the condition of the palatal bone. Cone beam computed tomographic scans should be used in future studies to better understand what is happening around these implants, especially regarding the regenerated bone in their palatal aspect.

## Conclusions

Despite its limitations (retrospective nonrandomized design and limited sample) the outcomes of the present study suggest that palatal positioned implants may be a good treatment alternative for patients with severe horizontal maxillary alveolar bone atrophy. Palatal positioned implants presented similar success rates, soft tissue conditions and peri-implant bone loss than well-centered implants placed completely surrounded by bone in non-atrophic ridges.

More studies with larger samples and a prospective, randomized controlled design are necessary to confirm these findings and to compare this technique with other alternatives for the rehabilitation of atrophic maxillae.
